# Validation of cardiovascular risk prediction in Type 2 diabetes through federated cohorts in Europe

**DOI:** 10.1093/eurpub/ckac131.580

**Published:** 2022-10-25

**Authors:** F Carinci, L Pennells, S Kaptoge, C Petitjean, S Gualdi, M Massi Benedetti, E Di Angelantonio

**Affiliations:** 1 Department of Statistical Sciences, University of Bologna, Bologna, Italy; 2 Department of Public Health and Primary Care, University of Cambridge, Cambridge, UK; 3 Internet Express snc, Pescara, Italy; 4 Hub for International Health ReSearch, Perugia, Italy

## Abstract

**Background:**

The SCORE2 risk model has been recommended for cardiovascular risk assessment in individuals aged over 40 years without diabetes in 4 defined risk regions of Europe. We aimed to validate a novel SCORE2-DM model in Type 2 diabetes with additional risk factors mainly based on UK datasets, using federated databases from the EUBIROD network.

**Methods:**

We defined a full operational protocol to implement a standard procedure for validation across contributing sources in Europe. The manual described inclusion/exclusion criteria (aged 40+, diagnosis of T2 diabetes at 30+, no prior CVD), risk factors measured over a baseline interval (1/2013-62015), target/competing events at follow-up (2015-2019). We specified a common data model with 9 steps required to process longitudinal records and deliver summary cohort data (one record per person). All rules were implemented in R and NeuBIRO, an original tool written in Java/Groovy and H2 SQL (https://github.com/eubirodnetwork/neubiro).

**Results:**

Software was able to produce the following outputs at each source: distribution of risk factors by sex and 5-year age groups; Harrell's C-index and standard error of SCORE2 and SCORE2-DM by sex and 10-year age groups, C-index differences; average 5-year predictive vs observed risk by risk deciles; adjusted cumulative incidence of 5-year competing risk by sex and 5-year age groups. Code was packaged into a stand-alone bundle, with test data and coefficients ofthe SCORE2-DM model. The procedures allowed either creating cohort data autonomously to run the supplied R code, or let NeuBIRO complete all steps foreseen to deliver. Tests have been successfully completed in the derivation data, with results from federated databases expected to contribute to the final external validation of SCORE2-DM by midyear.

**Conclusions:**

We defined a collaborative method to validate risk prediction models in high risk subgroups using international pooling of cohort data with privacy protection.

**Key messages:**

• Collaborative methods to validate risk prediction models can enhance access to real world data for researchers across chronic diseases.

• Implementation of flexible and reusable source code can increase opportunities to use prediction models for robust clinical decision making in multimorbidity.

